# Recruitment Dynamics of the Relict Palm, *Jubaea chilensis*: Intricate and Pervasive Effects of Invasive Herbivores and Nurse Shrubs in Central Chile

**DOI:** 10.1371/journal.pone.0133559

**Published:** 2015-07-28

**Authors:** Marina Fleury, Wara Marcelo, Rodrigo A. Vásquez, Luis Alberto González, Ramiro O. Bustamante

**Affiliations:** 1 Facultad de Ciencias Forestales y de la Conservación de la Naturaleza, Universidad de Chile, Santiago, Chile; 2 Instituto de Ecología y Biodiversidad, Departamento de Ciencias Ecológicas, Universidad de Chile, Santiago, Chile; Institute of Genetics and Developmental Biology, Chinese Academy of Sciences, CHINA

## Abstract

Shrubs can have a net positive effect on the recruitment of other species, especially relict species in dry-stressful conditions. We tested the effects of nurse shrubs and herbivory defoliation on performance (survival and growth) of nursery-grown seedlings of the largest living palm, the relict wine palm *Jubaea chilensis*. During an 18-month period, a total of more than 300 seedlings were exposed to of four possible scenarios produced by independently weakening the effects of nurse shrubs and browsers. The experiment followed a two-way fully factorial design. We found consistent differences in survival between protected and unprotected seedlings (27.5% and 0.7%, respectively), and herbivory had a dramatic and overwhelmingly negative effect on seedling survival. The invasive rabbit (*Oryctolagus cuniculus*) is clearly creating a critical bottleneck in the regeneration process and might, therefore, partially explain the general lack of natural regeneration of wine palms under natural conditions. Apparently biotic filters mediated by ecological interactions are more relevant in the early stages of recruitment than abiotic, at least in invaded sites of central Chile. Our data reveal that plant-plant facilitation relationship may be modulated by plant-animal interactions, specifically by herbivory, a common and widespread ecological interaction in arid and semi-arid environments whose role has been frequently neglected. Treatments that protect young wine palm seedlings are mandatory to enable the seedlings to attain a height at which shoots are no longer vulnerable to browsing. Such protection is an essential first step toward the conservation and reintroduction of this emblematic and threatened species.

## Introduction

In arid and semi-arid environments, environmental gradients differ at local scales because shrubs generally improve soil fertility and produce microclimates under their canopies that favor plant-plant interactions [1]. According to the stress-gradient hypothesis (SGH) [2], interactions among plants vary with environmental conditions, shifting from competition to facilitation as environmental stress increases. Such environmental stress comprises stresses from abiotic and biotic (i.e., consumers) sources. It has been found that plant seedlings growing beneath shrubs have different probabilities of survival than do conspecific seedlings growing in open interspaces between shrubs [3].

The occurrence of facilitation interactions under high abiotic stress conditions has been verified by numerous studies in many ecosystems and has provided a crucial basis for empirical studies and theory governing plant-plant interactions in stress-prone Mediterranean environments (see [1] and references therein, [3]) where tree seedlings benefit from habitat amelioration, especially during summer drought [3–9]. However, information on biotic impacts caused by consumers in these environments is still scarce (see [10] and references therein). Positive plant-plant interactions should be especially important in plant communities subjected to both climatic stress and biotic stress [2], as is the case in Mediterranean-type ecosystems. Surprisingly, the role of herbivory, a common and widespread interaction in arid and semiarid environment, has attracted little attention in this context [11–14].

In Mediterranean central Chile, the exotic mammalian herbivores have been altering the structure and functioning of ecosystems [15] by, promoting homogenization of species like *Acacia caven* and preventing the recolonization of clearings by woody matorral species on the slopes [8, 15]. It is expected that the impacts of introduced herbivores on native vegetation will be different from the native fauna [16], but how plant-plant facilitation relationship may be modulated by animal-plant interactions, specifically in this case, by herbivory, has been frequently neglected.

Valiente-Banuet, Rumebe, [17] have indicated that facilitation (or the nurse effect) prevented Tertiary species extinction when climatic conditions became more xeric during the Quaternary, the period during which the current Mediterranean climate emerged. This effect should be of particular importance for the persistence of fleshy-fruited Tertiary species with recalcitrant seeds [17] as palms (Arecaceae). Originating in the Cretaceous, palms experienced a spectacular radiation in the Tertiary Early Cenozoic and severe extinction rates due to rainforest decline during the drier Quaternary [18]. Thus, relict palms in arid ecosystems, such as the endemic wine palm (*Jubaea chilensis* (Molina) Baill.) in Mediterranean central Chile, constitute a proper candidate for evaluating the relative importance of abiotic and biotic (herbivory) stresses for seedling survival and growth, and for assessing the relationship between animal-plant and plant-plant interactions in arid environments.

Accordingly, we formulated four hypotheses (i) Shrub canopies, by creating a more humid, mesic, and shaded environment relative to open interspaces, differentially affect palm seedling performance. (ii) Shrubs interact positively with wine palm seedlings to facilitate recruitment, buffering the seedlings performance from potentially limiting stresses (SGH) or (iii) seedling performance is facilitated because shrubs protect the seedlings against browsers. In order to study these hypotheses, we monitored growth and herbivore defoliation of 300 wine palm seedlings over a period of 18 months. Seedlings were transplanted outside and under the canopy of the seven most representative shrub species and were grown with and without exclosure protection from browsing. This study can improve our understanding of the relative importance of biotic and abiotic mechanisms for shrub facilitation in the Mediterranean hotspot ecosystem region, which has experienced an increasing frequency, duration, and severity of drought in recent decades [19] and has also faced risks associated with the presence of a high proportion of invasive non-native vertebrate herbivores [8, 15, 20]. Also, this study will help us to examine to fate of wine palm in a context of increasing of aridity and herbivory pressure.

## Materials and Methods

### Study species and site

This study was conducted in the Oasis La Campana private reserve in central Chile (hereafter La Campana; 71°04'W; 32°57’S). The CONAF/Parque Nacional la Campana and Reserva Oasis de La Campana issued all of the required permits for the work conducted in those sites. Located in the core zone of the Campana-Peñuelas UNESCO Biosphere Reserve, La Campana covers 17,095 ha and has irregular topography varying from 300 to 1,800 m altitude. The area has a Mediterranean climate, closely equivalent to that of southern California but with a six-month offset. The mean annual rainfall and temperature are 109 mm and 20μC, respectively [21]. Rainfall is concentrated in spring and autumn, alternating with hot and dry summers and cold winters. Mean temperatures for December-February are 18°C, and a mean of only 30 mm of precipitation falls from November to April. Winters are mild and moist, and the mean winter temperature, from June to August, is 11°C [22].

Mediterranean-type ecosystems have been substantially altered by human activities worldwide, but it is probable that such ecosystems persist to a greater extent in central Chile than in other regions of the world [23]. The natural recovery of sclerophyllous vegetation in many open and abandoned areas of central Chile is a slow process [24, 25] due to unsuitable conditions for seedling recruitment and a lack of seed sources. The limiting factors for recruitment include seed deposition in unsafe sites for seed germination and for seedling establishment [24, 25].

**Fig 1:**
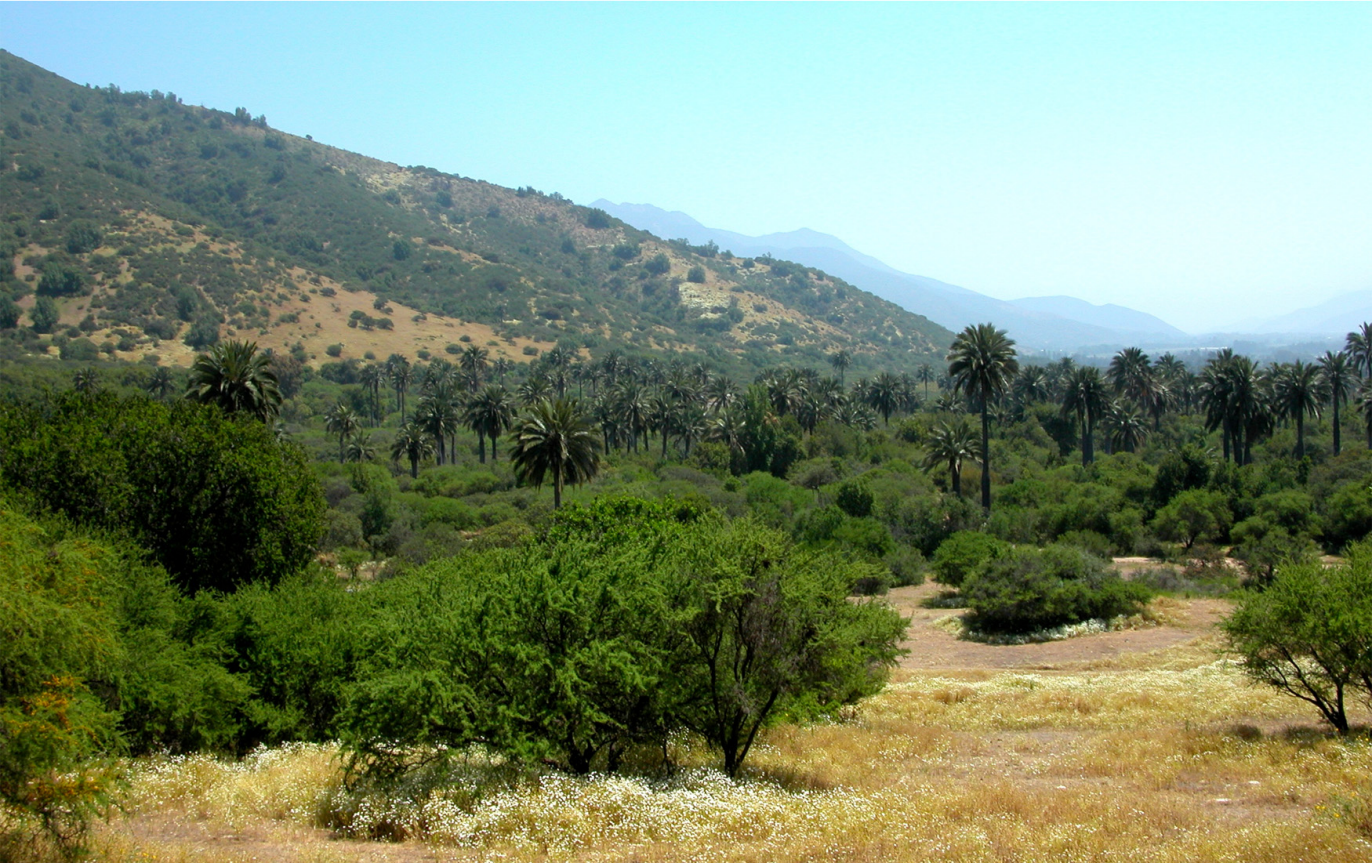
Palm forests in La Campana National Park, central Chile.

The most common evergreen trees and shrubs in La Campana are *Cryptocarya alba* (Lauraceae), *Lithraea caustica* (Anacardiaceae), *Quillaja saponaria* (Quillajaceae), *Maytenus boaria* (Celastraceae), *Kageneckia oblonga* (Rosaceae), and *Peumus boldus* (Monimiaceae) [26]. Sclerophyllous forests commonly exhibit a patchy spatial structure, but they may present a continuous canopy in ravines, deep creeks, along permanent water courses and on south-facing slopes [27]. On dry north-facing slopes or in frequently disturbed sites [28], the sclerophyllous vegetation is often replaced by a xerophytic thorn scrub, with a combination of deciduous shrubs, such as *Retanilla trinervia* (Rhamnaceae), *Colliguaja odorifera* (Euphorbiaceae), *Baccharis linearis* (Compositae), *Acacia caven* (Leguminosae) and *Puya berteroniana* (Bromeliaceae) [26]. The palm forest, where this study was conducted, is narrowly distributed within native vegetation and frequently neglected component with numerically abundant populations of the endemic wine palm *Jubaea chilensis* (Molina) Baill (Fig 1). The wine palm population has been severely fragmented and currently occurs mainly on slopes and in deep canyons of the central Chile Coastal Range [29]. It is probable that these sites, with soils of granitic origin, are favorable because of their maritime, moisture-laden air and limited temperature oscillations [23].

La Campana, the heart of the wine palm's range, contains the world’s largest remnant population of the endemic wine palm, harboring ≈ 60,000 wine palms that represent more than 58% of the total number of native stands [29]. The wine palm has a limited distribution but is numerically abundant where it is found (22–113 ind. ha^-1^) [30]. It is usually dominant in its favored habitat of slopes and deep canyons with moisture-laden marine air and limited temperature oscillations [27].

In central Chile, the biota has been widely and severely impacted by agriculture, human-caused fires, and livestock grazing [22], activities that still persist in areas surrounding the study site [31]. Introduced species of seedling predators are commonly found within the area. These species, including the invasive European rabbit (*Oryctolagus cuniculus*) [32] and livestock [33], have potential negative effects on natural regeneration. The study area also holds most of the Mediterranean-type vegetation of central Chile, including hygrophyllous and sclerophyllous forests, *Nothofagus* trees and palm forests [31].

The wine palm can attain a height of 25 to 30 m and a trunk diameter of up to 180 cm. The distinctive trunk is usually bulb shaped, with the thickest portion of the trunk above the base of the plant. *J*. *chilensis* produces prolific ovoid fruits in the form of yellow-orange drupes; within each drupe there is a single spherical fruit that is approximately 2–3 cm (0.79–1.18 in) in diameter. The fruit has a very hard endocarp (shell) and a whitish endosperm. The fresh nuts are commonly sold in the areas where the palms grow during their fruiting season (L.A. González, pers. comm.).

### Experimental design

During the fruiting season of 2004, wine palm seeds were collected from several palm trees. For more than 1,000 individual seeds, seed mass was recorded and the seeds sown in an adjacent nursery cubicle (Reserva Oasis de La Campana). Seedlings were grown from early to late January 2005.

Five months later, more than 300 wine palm seedlings were randomly selected, with initial seedling aerial biomass weighing 0.94 ± 0.02 g (mean ± SE, n = 300). Because the distribution of palms is strongly influenced by the microhabitats within their range [30, 34], which are distributed non-randomly [27], three areas with suitable wine palm populations were selected. The conditions of the physical environment under shrub canopies and interspaces between shrubs differ due to variations in vegetation cover (open interspaces between shrubs 7.67 ± 1.36%; under shrub 63.33 ± 1.27%, n = 150) and moisture soils (open interspaces between shrubs 3.23 ± 0.22%; under shrub 5.25 ± 0.22% moisture content), especially during hot, dry summers. These differences can be assumed to result in distinct microhabitats [35]. The 75 seedling-performance experimental stations were simultaneously established at minimum distance of 50 m intervals. Seedling-performance experimental stations were spatially independent from another such that we did not detect spatial autocorrelation in seedling survival or growth among stations (average Moran’s I-values were not significant for all distance classes).

Each station consisted of four wine palm seedlings and a native shrub of over 1 m of tall, that completely shading the ground surface bellows its crown. Although using different nurse shrub species could cause potential confounding factors, five shrub species have been used due to the species presence and abundance: *A*. *caven*, *R*. *trinervia*, *L*. *caustica*, *B*. *linearis*, *Q*. *saponaria*, *Azara* sp. and *P*. *boldus*, thus ranging a wide spectrum of nurse life forms and architecture. While we cannot altogether discount the possibility of shrub species interacting on herbivory patterns or soil type patterns, our preliminary analysis found no difference between shrub species and wine palm seedling survival (General Linear Model, F = 0.62, df = 6, P = 0.71). In addition, previous studies have shown that the phylogenetic relationship of plants is a weak predictor of the similarity of associated herbivore assemblages, and it may not influence herbivore behavior [36].

**Fig 2:**
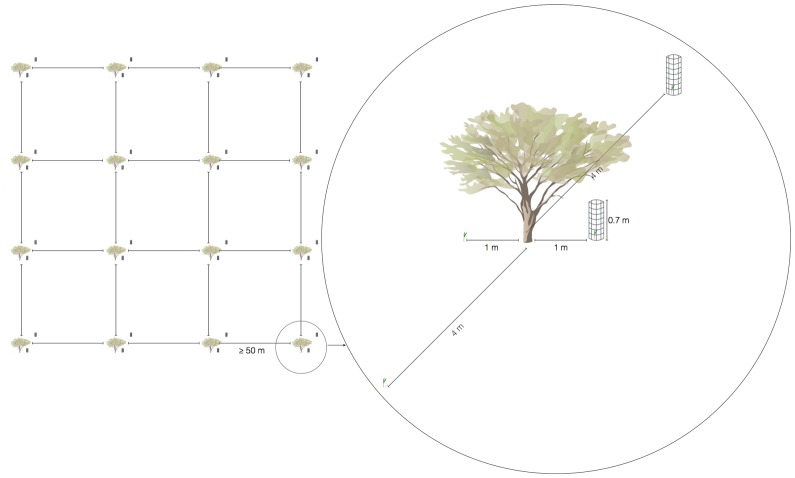
Schematic of plot designs, with shrubs indicating the 75 experimental seedling-performance stations established at minimum distance of 50 m intervals. In detail, the planting arrangement of four wine palm seedlings in a factorial design.

**Fig 3:**
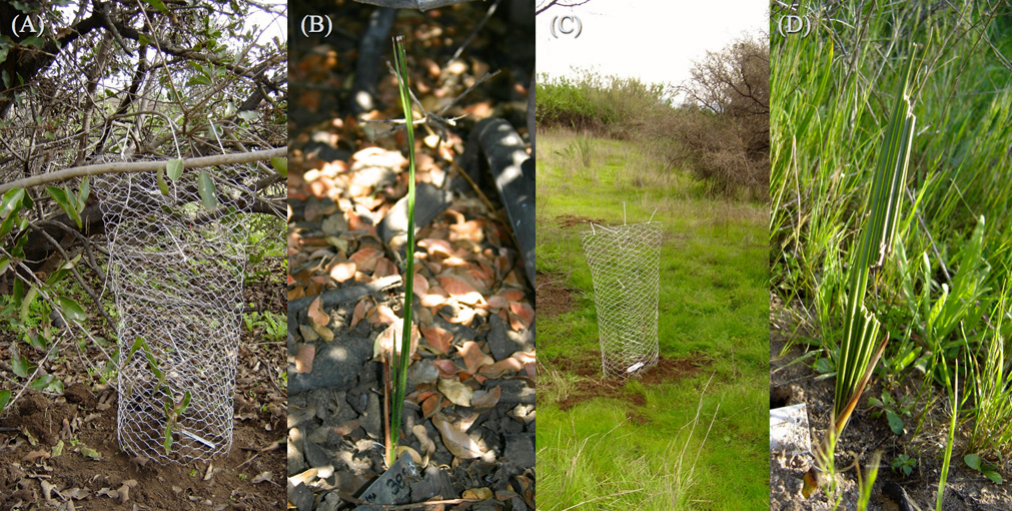
Herbivore-nurse shrub factorial experiments used to quantify seedling performance. Where (A) browsers excluded but seedlings under shrub cover; (B) exposed to browsers but under shrub cover; (C) browsers excluded but seedlings without the protection of a shrub canopy; and (D) fully exposed plants.

A two-way fully factorial design was used for the study. The seedlings experienced one of four possible selective regimes generated by selectively weakening the effects of browsers and nurse shrubs independently (Fig 2). The study design involved the following four combinations of factors: (i) browsers excluded but seedlings under shrub cover; plants were surrounded by a cylinder of wire netting with 1/2-inch mesh openings with a height of 70 cm and buried at least 20 cm deep to exclude browsers, planted under a shrub canopy directly beneath the zone of maximum canopy cover of the most common shrub species (Fig 3A); (ii) exposed to browsers but under shrub cover, seedlings unfenced to allow herbivore access and planted under the canopy of the most common shrubs (Fig 3B); (iii) browsers excluded but seedlings without the protection of a shrub canopy, plants surrounded with a 70-cm-high cylinder of wire netting that hampered access by browsers and lacked the potential facilitative effect of nurse plants (Fig 3C); and (iv) fully exposed plants, unfenced seedlings planted interspaces between shrubs (Fig 3D).

The seedlings were surveyed every four weeks during the first third of the year-long experiment (July 2005 to March 2006) and once in summer (January 2007); hence, 11 sets of measurements were obtained. At each census, all seedlings were identified as alive or dead, distinguishing between abiotic and biotic stress (i.e., dried by stressful environmental conditions or consumed by herbivores); the number of shoots and shoot length individually measured; and examined for herbivore damage, expressed as the difference between the length of an affected shoot and the length previously measured. Two herbivore species, the exotic European rabbit (*Oryctolagus cuniculus*) and the endemic rodent degu (*Octodon degus*) were assessed, as both species usually defecate very close to the areas where they eat [32]. Domestic livestock were excluded from the experimental areas.

### Data analysis

The temporal patterns of seedling death were addressed to compare seedling survival among the browser and microhabitat treatments. Curves of seedling survival over time were generated using Kaplan-Meier estimates [37, 38]. The survival functions describe the probability that an individual survives longer than a specified period, considering individuals at risk at the beginning of each interval, i.e., the lapse between two mortality events, and excluding censored values. Browser exclusion, microhabitat, and shrub type were introduced as factors [38]. A log rank (Mantel-Cox) test [39] was used to compare pairs of survival functions. The consequences of microhabitat, browsers, and their interaction for *J*. *chilensis* seedlings were assessed by fitting General Linear Mixed Models (GLMMs) separately to the seedling survival and seedling growth data for 2005–2007. The dependent variable in the models was the number of seedlings surviving in each plot or the difference in seedling biomass from the beginning on the experiment to the end. Exclusion from browsers and microhabitat and two-way interactions were inserted in the models as fixed effects. Time was included in the ‘repeated’ statement, where the scores were nested within individuals. Computations were performed with the SAS MIXED procedure and restricted maximum likelihood estimation [40]. A Pearson correlation coefficient was used to quantify the relationship between seed size and log-transformed seedling growth. We tested the data for conformity with the assumption of homogeneous variances. All comparisons reported used two-tailed significance tests at the 0.05 level and were performed with R [41] except for GLMMs fitted with the PROC MIXED package of SAS version 9.1 (SAS Institute Inc., Cary, NC). All relevant data are presented in S1 Table.

## Results

Our results show that for *Jubaea chilensis*, seedling survival and growth were not affected by seed size (Pearson correlation, P > 0.05 for both comparisons). In fact, no differences in seedling growth were observed among treatment groups. The mean seedling growth was 0.94 ± 0.02 g and did not vary according to the microhabitat, shrub type or herbivore access (General Linear Mixed Model, P > 0.05 for all comparisons, Table 1). However, survival depended heavily on protection from vertebrate herbivores. By the end of the 18-mo period, 42 (14%) of the 300 seedlings remained alive, with 109 affected by abiotic unsuitable conditions (dried seedlings) and 149 lethally attacked by vertebrates.

**Table 1:** General linear mixed model effects.

Atribute	Treatment	*F*	*df*	*P*
Survival	Brownser access × nursing effect	21.43	1	0.00
	Nursing effect	3.86	1	0.05
	Brownser access	488.19	1	0.00
Growth	Brownser access × nursing effect	1.65	1	0.20
	Nursing effect	0.38	1	0.54
	Brownser access	1.65	1	0.20

**Fig 4:**
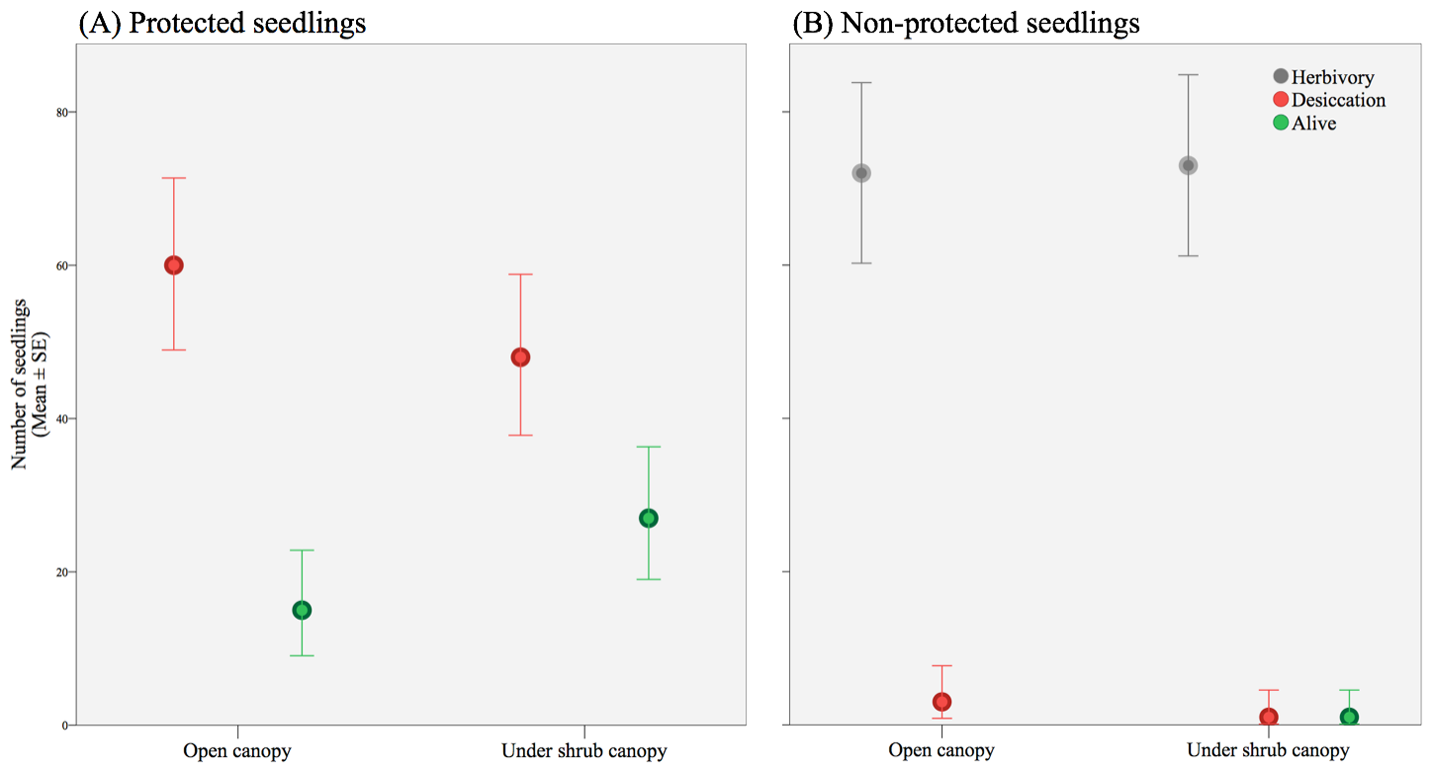
Average wine palm seedling mortality caused by ‘desiccation’ (when it was dried) or ‘herbivory’ (when damaged, broken, or chewed stems or leaves). Death and survival (‘alive’) rates were scored after 18-month study period to (A) protected and (B) non-protected seedlings to browsers. Error bars indicate the standard error (SE) of the mean.

**Fig 5:**
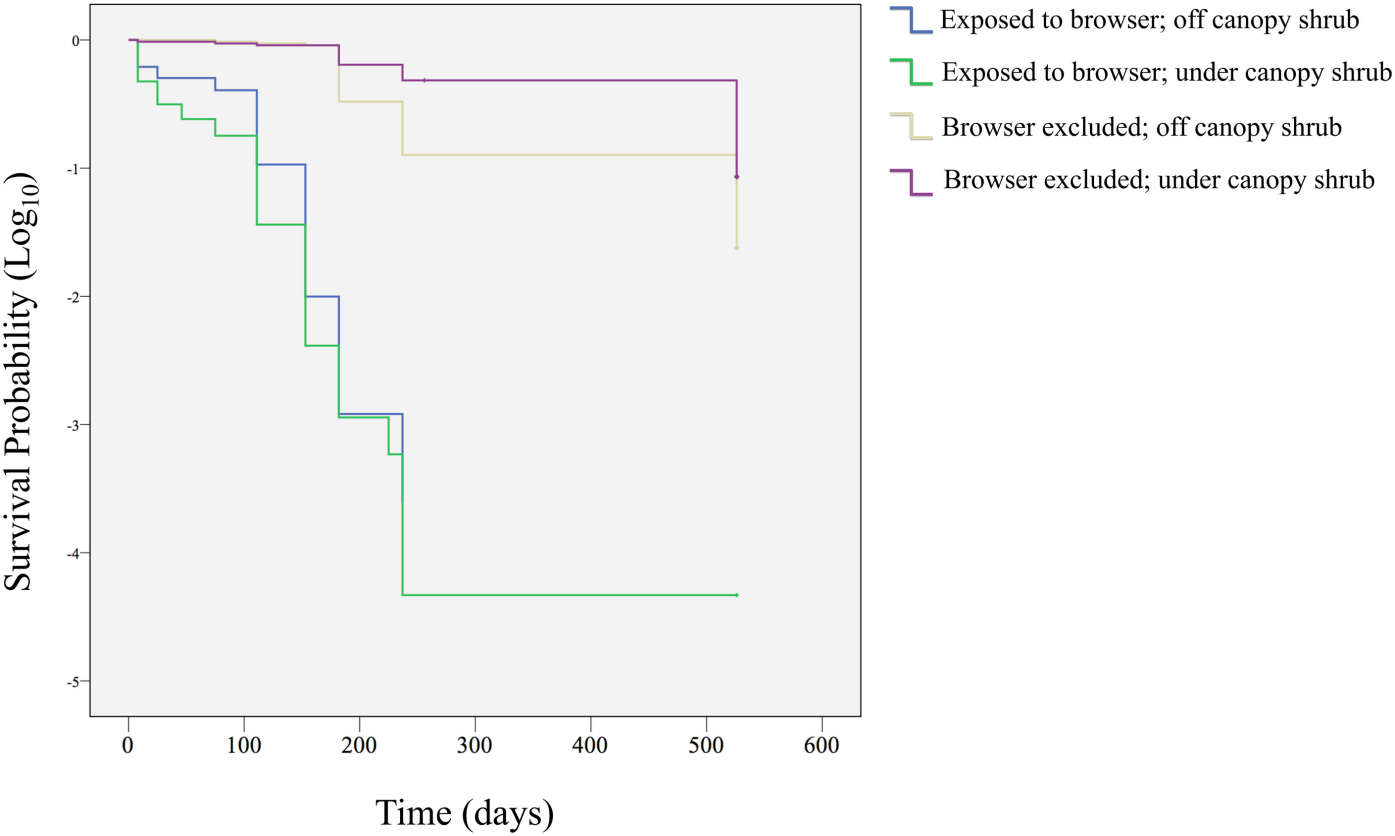
The functions were calculated for an 18-month study period to compare wine palm seedling survival among treatments.

The seedlings that were accessible to browsers suffered higher mortality rates than those that were enclosed and inaccessible to vertebrates (F = 488.19, df = 1, P < 0.0001, Table 1). Regardless of microhabitat (outside and under shrub canopy), unfenced seedlings had negligible survival (n = 1, Figs 4 and 5), with more than 95% of deaths evidently produced by biotic stress and a minor proportion triggered by abiotic stress (1.3% under canopy shrubs; 3.5% open canopy), showing that shrubs do not provide protection against herbivores as seedlings showed the same survival probability when exposed to browsers in the open interspaces between shrubs and under shrubs. However, an interaction between microhabitat and seedling protection was observed for seedling survival (F = 21.43, df = 1, P < 0.0001, Table 1), with better performance of fenced seedlings under shrub canopies (Fig 4).

The probability of seedlings remaining alive differed significantly between treatments (Fig 5), with earlier deaths in wine palm seedlings that were exposed to herbivores. Fenced seedlings had an estimated mean time (± SE) to failure of 331 ± 19 and 433 ± 18 days for open interspaces between shrubs and growth under shrub canopy, respectively, much longer than the mean time predicted for seedlings accessible to browsers (under canopy = 85 ± 9 days; fully exposed seedlings = 114 ± 11 days). The proportion of caged seedlings died under the shade of shrubs canopies was lower than those exposed to browsers attack but under shrub cover (log-rank, χ^2^ = 128.34, P < 0.0001), or than those inaccessible to browsers but seedlings without the protection of a shrub canopy (χ^2^ = 10.20, P = 0.001) or than those fully exposed seedlings, i.e., unfenced seedlings planted in open interspaces between shrubs (χ^2^ = 118.99, P < 0.0001). In fact, caged and unshaded wine palm seedlings survived longer than those under shrub canopies exposed to browsers (χ^2^ = 102.41, P < 0.0001) and or than those fully exposed seedlings (χ^2^ = 116.24, P < 0.0001). However, the survival of wine palm seedlings was not influenced by nurse shrub protection on those seedlings open to browser (χ^2^ = 3.5, P > 0.05, Fig 4).

## Discussion

Our findings from experimentally decoupling the herbivory and abiotic effects on wine palm seedling performance support the scarce empirical evidence indicating that semi-arid ecosystems have a strong impact on plant-plant interactions [11, 13, 14]. The intensity of shade-induced facilitation of growth did not differ during the course of the experiment, whereas its effect on survival did vary. The effects of shrub interactions on seedling survival differ from their effects on growth have been previously observed [42, 43], and that the intensity of facilitation of growth by shade did not vary with adverse environmental conditions may be consistent with other studies [42, 44, 45]. Once wine palm seedlings are able to escape from herbivores, the facilitative effect on modifying environmental conditions seem to null or at least reduce as seedlings showed similar growth in the ameliorated conditions under the shrubs and in the harsh open canopy. The effects of facilitation on survival and growth may vary simultaneously, probably as a consequence of differences in environmental requirements according to the plant life-stage.

Desiccation during the summer and consumption by vertebrate herbivores, most likely due to the introduced and invasive European rabbit (*Oryctolagus cuniculus*), are important sources of wine palm seedling mortality. Contrary to our hypothesis, herbivory, rather than harsh environmental conditions, is the strongest inhibiting effect on *Jubaea* seedling establishment. Other studies have also shown that biotic filters mediated by animal-plant interactions might be more important than abiotic filters in the early life stages of plants [46–48]. Nevertheless, in the absence of consumer pressure, the abiotic mechanism of facilitation by nurse shrubs was observed for survival but not for seedling growth.

Our findings provide only partial support for the SGH [2], as facilitation did not increase seedling survival or growth under high herbivore pressure (biotic stress). However, seedling establishment and growth depend presumably on the combined effect of biotic and abiotic filters and shrubs cannot provide the facilitative effects of abiotic stress amelioration and protection against herbivores. Thus, under extreme conditions, survival may not be possible even in the presence of positive interactions because shrubs do not facilitate seedling performance by providing protection against browsers, the primary and pervasive constraint to *Jubaea* seedling establishment. In contrast, at intermediate or low levels of herbivory, the wine palm seedlings are able to overcome mortality due to herbivore consumption and higher survival of shaded plants is a result of facilitation provided by shrubs due to the creation of a more humid, mesic and shaded environment, especially during summer, thus supporting the SGH. However, at high stress levels, with elevated consumer pressure, the net positive effect disappears. These results concur with the predictions Smit, Rietkerk [10] corroborated by Saiz and Alados [13], Graff and Aguiar [49], and Graff, Aguiar [50].

In low-productivity sites such as semi-arid Mediterranean habitats, stress-adapted herbivore species appear to compensate for nutritionally poor food by increasing the content of their diet, changing their foraging behavior by tending to be less selective, and increasing their feeding area [49, 51, 52]. Fuentes, Jaksic [32] demonstrated that in the central Chile, the European rabbit (*Oryctolagus cuniculus*) and the degu (*Octodon degus*), an endemic rodent, show differential killing effects on native vegetation, with the rabbits foraging over wide areas while the degu restricting its search activity to an area a few meters from their refuges. Jaksic, Fuentes, [53] add that kitten and juvenile rabbits are ecologically comparable to degu, limiting their foraging to areas under the canopy [16] possibility for explaining the observed widespread high mortality of unfenced wine palm seedlings and weakening the facilitative effect of shrub canopies.

From a conservation and management perspective, small invasive consumers such as the alien rabbit play a major role in shaping vegetation dynamics, with consequences for biodiversity worldwide ([54], see [55]). This form of herbivory is one of the main causes of the lack of seedlings in stands of the endangered *J*. *chilensis*, a relict coming from the Tertiary Early Cenozoic that resisted even severe extinction rate due to rainforest decline in the Quaternary. Thus, the sensitivity of how plant-plant facilitation might be affected to perturbation could cause important disruptions in ecosystems modifying the species recruitment patterns. In extreme cases, herbivore overbrowsing can have profound effects on ecosystem processes through overconsumption, effectively reducing the diversity and carrying capacity for consumers and potentially leading to extinction or even to ecological meltdown [56, 57].

Our results show that despite the facilitation effect that nursing shrubs have on wine palm seedling establishment, recolonization of open spaces is possible without the facilitation of a nurse shrub, but protection of young wine palm seedlings is obligatory to enable them to attain a height at which shoots are no longer vulnerable to browsing.

Our findings also stress the importance of assessing the influence of sources of biotic stress, specifically herbivory, on plant interactions as part of the effective planning for the preservation, restoration and management of arid and semi-arid ecosystems. Invasive vertebrate herbivores in natural ecosystems has being recorded all over the world (e.g. [57], [58], [59–63]) once both fragmented and continuous landscapes are suffering from the human-induced disturbances, with strong implications for the persistence of key ecosystem processes. We highlight the importance of assessing the impact of invasive mammals on ecosystems, as the invaders might limit seedling establishment not only for the studied species but other plant species, as well as affecting all steps of the recruitment process, with ensuing impacts on the population dynamics of the affected plants.

## Supporting Information

S1 Table Wine palm seedling survival and growth among treatments. (XLSX)
